# Evaluation of acute oral toxicity of *Ipomoea turpethum* extract loaded polymeric nanoparticles in Wistar rats

**DOI:** 10.3389/fphar.2023.1086581

**Published:** 2023-03-15

**Authors:** Sanskriti Swami, Mohd Mughees, Sana Kauser, Saima Wajid

**Affiliations:** Department of Biotechnology, School of Chemical and Life Sciences, Jamia Hamdard, New Delhi, India

**Keywords:** polymeric nanoparticle, acute oral toxicity, drug delivery system, FT-IR, gas chromatography-mass spectrometry

## Abstract

**Introduction:** The amalgamation of novel drug delivery techniques and potential drugs is considered the most promising tool for the treatment of diseases. In our study, we have employed N-isopropyl acrylamide, N-vinyl pyrrolidone, and acrylic acid (NIPAAM-VP-AA) copolymeric nanoparticles for delivering *Ipomoea turpethum* root extract. *I. turpethum* is a perennial herb (Convolvulaceae family) and has been used as medicine for ages. The present study was conducted to evaluate the safety of *I. turpethum* root extract-loaded NIPAAM-VP-AA polymeric nanoparticles (NVA-IT) in Wistar rats.

**Methods:** An acute oral toxicity study was conducted in accordance with OECD guidelines 423 for the testing of chemicals. Different doses of NVA-IT i.e., 5 mg/kg, 50 mg/kg, 300 mg/kg, and 2000 mg/kg were administered to female Wistar rats in a stepwise manner using oral gavage. The toxicity signs were thoroughly observed for the next 14 days. At the end of the study, the blood and vital organs were harvested for hematological, biochemical, and histopathological studies.

**Result:** No mortality or pathological anomalies were observed even at the highest dose which exemplifies that the lethal dose would be more than 2000 mg/kg body weight (GSH category 5). Behavioral changes, biochemical parameters, and histopathology of vital organs were normal after NVA-IT administration.

**Conclusion:** This study demonstrated that NVA-IT nanoparticles are non-toxic and can be considered for therapeutic use in different diseases, such as inflammation, CNS diseases, Cancer, etc.

## 1 Introduction

*Ipomoea turpethum* or *Operculina turpethum* is a plant widely known for its medicinal properties in both the Unani and Ayurvedic medicine systems. It is globally distributed, known by different names including “Trivrit” in Ayurveda, “Turbad” in Persian, “India Jalap/turpeth” in English, and plenty more. Belonging to the Convolvulaceae family, the plant is scattered in Asian countries like India, China, Sri Lanka, the Philippines, Pakistan, and Bangladesh, as well as the America and Africa (Kohli et al., 2010; Gupta and Ved, 2017).

Since ancient times, *Ipomoea turpethum* has been used to cure and relieve the symptoms of many diseases. Ailments like fever, edema, asthma, hemorrhoids, dyspepsia, flatulence, gout, paralysis, abdominal tumors, and worm infestation had been cured by the internal administration of the plant parts, especially the roots. Trivrit root paste, powder, and oil are reportedly beneficial for skin diseases, snake and scorpion bites, fistula, cervical lymphadenitis, hemorrhoids, tuberculosis, and herpes (Sharma and Singh, 2012). A gas chromatography-mass spectrometry (GC-MS) study of the plant reported 71 active compounds present in the root extract (Choudhary et al., 2020). The active compounds found in this study correlate with the plant’s ancient use; for instance, D-arabinose shows anthelminthic properties, and alpha-linolenic acid has cardioprotective and anti-inflammatory properties (Stark et al., 2001; Sakoguchi et al., 2016). Moreover, active compounds such as cinnamic acid, succinic acid, valeric acid, palmitic acid, saponins, coumarin, lupeol, stigmasterol, and ß-sitosterol are well-known for their anticancer properties. Recent studies have shown the direct impact of *I. turpethum* extract on different types of cancer cell lines such as oral and breast cancer (Arora et al., 2017; Mughees and Wajid, 2020).

Nanotechnology, as a fast-evolving field, offers exciting possibilities for the early detection and therapy of various diseases. Nanoparticle-based formulations bypass biological barriers, allowing for longer blood circulation, site-specific targeting, and reduced toxicity (Liyanage et al., 2019; Zubair et al., 2021). These formulations have a higher therapeutic efficiency than traditional treatment due to their better specificity for effective drug targeting and lack of solubility and stability issues. Formulated in varied sizes and materials (organic material like lipids and inorganic materials like gold and polymers), nanoparticles as nanocarriers have gained interest from every discipline of medicine owing to their capacity to transport drugs in the optimal dose range, which contributes to increased therapeutic efficiency and decreased adverse effects (Amoabediny et al., 2018; Jin et al., 2020).

In the present study, we used copolymerized N-isopropyl acrylamide (NIPAAM), vinyl pyrrolidone (VP), and acrylic acid (AA) nanoparticles loaded with the root extract of *I. turpethum*. NIPAAM co-polymerized with vinyl pyrrolidone and acrylic acid makes it double triggered; thus, it collapses in response to changes in temperature and pH (Ahmad et al., 2013; Ali et al., 2016; Mughees et al., 2019).

This study tracked the therapeutic safety of *I. turpethum* root extract-loaded nanoparticles *in vivo* through an acute oral toxicity study in female Wistar rats. The study was divided into three parts: 1) synthesis and characterization of NIPAAM-VP-AA polymeric nanoparticles, 2) identification of different compounds present in the ethanolic root extract of the *I. turpethum* plant, and 3) study of the acute oral toxicity of *I. turpethum* root extract-loaded nanoparticles in female Wistar rats. All animal experiments were conducted according to the OECD Guidelines for the Testing of Chemicals (No. 423), Acute Oral Toxicity—Acute Toxic Class Method with slight modifications.

## 2 Materials and methods

### 2.1 Plant material and extract formation

*I. turpethum* plant material was collected from the Foundation of Revitalization of Local Health Traditions (FRLHT, Bangalore, India). The sample was further identified by the expert Dr. Sunita Garg (Head, Raw Materials Herbarium & Museum, CSIR- NISCAIR, New Delhi, India) with reference number **NISCAIR/RHMD/Consult/2015/2826/19**. The sterilized root of the plant was shade-dried and powdered to prepare the ethanolic extract in which 5 g of root powder was added to 20 ml of absolute ethanol and subjected to maceration in an incubator shaker for 24 h at 28°C with shaking at 100 rpm. The procedure was repeated for three consecutive days, with the 3-day filtrate collected in a round-bottom flask. The solvent from the filtrate was subjected to reflux at 45°C until 5 ml was left and then filtered (0.22-µm filter) and stored at 4°C for future requirements.

### 2.2 Nanoparticle preparation

The polymeric nanoparticles were synthesized using a free radical reaction in which the NIPAAM, VP, and AA monomers were combined in a molar ratio of 90:10:5 in double-distilled water. As per the detailed procedure, 180 mg of freshly recrystallized (with hexane) NIPAAM was mixed with 20 ml of double-distilled water followed by freshly distilled VP (20 µL) and AA (10 µL). Next, 150 µL of MBA (20 mg/ml) was used to cross-link the reaction, and the nitrogen gas was passed for 30–60 min to remove the dissolved oxygen from the reaction mixture. Then, 50 µL of FAS (5 mg/mL) and saturated APS each were added to initialize the polymerization. The reaction was kept in a 32°C water bath for 24 h to complete the polymerization. Once the polymerization was complete, the solution was subjected to dialysis *via* a cellulose dialyzing membrane (12 kDa cut-off) for 24 h followed by lyophilization to dry the sample for future use.

### 2.3 Characterization and measurement of the nanoparticle sizes and size distributions

#### 2.3.1 Dynamic light scattering (DLS)

Dynamic light scattering was used to measure the average size distribution using a Malvern instrument Zeta sizer Ver. 7.12 at 25°C. A lyophilized sample of the polymeric nanoparticle was completely dissolved in double-distilled water, and DLS measurements were taken. The Z-average and polydispersity index were calculated, and a size distribution by intensity graph was generated.

#### 2.3.2 Transmission electron microscopy (TEM)

To determine the external morphology of the polymeric nanoparticles, TEM imaging was performed using the TEM facility at the Sophisticated Analytical Instrumentation Facility (SAIF) at the All India Institute of Medical Science (AIIMS), New Delhi, India. A drop of water-dissolved lyophilized sample was placed on the carbon grid, and the excess was drained off. Next, the grid was placed in 2% phosphotungstic acid (PTA) for 2 min. The grid was dried at room temperature before observation by electron microscopy. The imaging was performed using a Tecnai G2, 20 S–twin, FEI Electron Optics, Holland-equipped digital imaging, and 35-mm photography system. The results were recorded as images acquired using a high-resolution digital CCD camera and processed using iTEM software (Olympus Soft Imaging System, Germany).

#### 2.3.3 Fourier-transform infrared spectroscopy (FT-IR)

The NIPAAM, VP, and AA monomers and polymerized micelles underwent FT-IR study on Varian 7000 FTIR and Varian 600UMA FTIR microscopes. All samples were grabbed on a KBr pellet before being placed on the FT-IR spectrophotometer. The data were analyzed using Origin 2021b software.

### 2.4 Drug loading

The ethanolic root extract of *I. turpethum* was loaded in the polymeric nanoparticle using the physical entrapment method. In this method, an aqueous solution of 40 mg of lyophilized powder was made by adding 20 ml of ddH_2_O. A diluted drug solution with a concentration of 10 mg/ml was made in ethanol from the stock solution of the plant extract. The extract was added to the solution with vigorous vortexing and minor sonication. Precipitate formation and haziness in the solution were considered signs of the completion of drug loading into the nanoparticles. The drug-loaded nanoparticle solution was further lyophilized for long-term storage and future use.

### 2.5 Gas chromatography-mass spectrometry (GC-MS)

GC-MS was performed on a Shimadzu QP-2010 Plus system with a Thermal Desorption System TD 20 and autosampler (AOC-20i + s). The injection needle was cleaned with pre-solvent four times and with solvent five times before injecting the sample (two times). For separation, an Equity-5 column measuring 30.0 m × 0.25 um × 0.25 mm, and helium gas as a carrier was used with a constant column flow of 1.21 mL/min. The initial column oven temperature was 80°C, with a hold time of 2 min, and was later increased in 10°C increments to 300°C, with a hold time of 16 min. The sample was injected at 260°C using a split ratio of 10.0. The ion source was heated at 220.00°C, and the interface temperature was kept at 270.00°C. The chromatogram spectra were recorded in the mass ranges of 40.00–650.00 m/z with a speed of 3333. WILEY8 spectral library and National Institute of Standards and Technology (NIST) database were used for peak identification and further confirmed by using ChEBI (http://www.ebi.ac.uk/chebi/init.do) and ChemSpider (http://www.chemspider.com/).

### 2.6 Animal study

A total of 15 female Wistar rats aged 6–7 weeks were procured from the Central Animal House Facility at Jamia Hamdard, New Delhi. The acute oral toxicity study was conducted according to the OECD 423 guidelines. The animals were acclimatized to laboratory conditions 1 week before starting the experiment. The animals were kept in standard laboratory conditions at a temperature of 20 ± 2°C, relative humidity of 50 ± 10%, and 12 h of dark and light cycles. The animals were provided food and water *ad libitum*. All experiments and procedures were performed according to the Institutional Animal Ethics Committee (IAEC) formed under the supervision of CPCSEA under the Ministry of Animal Welfare Division of the Government of India, New Delhi.

### 2.7 Experiment

The animals were grouped into five cages, with three animals per cage, and administered different doses in a stepwise manner: 5 mg/kg (Group A), 50 mg/kg (Group B), 300 mg/kg (Group C), 2000 mg/kg (Group D), and the vehicle (water, control). The animals fasted overnight before dosing. The dose was administered using stainless steel oral gavage, and the animals were provided free access to food 1 h after dosing. The experiment was initialized with the lowest dose, i.e., 5 mg/kg body weight, in three animals. As per OECD guideline 423, the absence or presence of mortality or moribund status of the animal in response to the compound in one step determines the next step. If no mortality occurs in the first step, the procedure is repeated for the next group of animals for the next higher dose. If all animals die, then the procedure is repeated at the next lower dose. The animals were kept under continuous observation for the first 30 min and then periodically for the next 24 h. Certain behavioral changes like appetite, diarrhea, motor activity, muscular weakness, piloerection, salivation, depression, and mood were noted (Adebiyi and Abatan, 2013). The animals were weighed before the dosing, and changes in weight were monitored weekly. The animals were observed daily for the next 14 days and humanely sacrificed on the 15th day of the experiment using CO_2_ inhalation after collecting blood from the tail vein. The blood was divided into two vials: one with EDTA for hematological study and the other without EDTA to separate the serum, which was further used for the analysis of biochemical parameters. Vital organs, including the heart, kidney, spleen, liver, brain, stomach, and intestine, were collected and stored in formalin for histopathological study.

### 2.8 Hematological study and biochemical parameters

Hematological parameters including hemoglobin, total RBC count, total WBC count, differential WBC count, and platelets were determined using a Sysmex XP-100 machine. The biochemical parameters including liver function (bilirubin, SGOT, SGPT, alkaline phosphatase, and total protein) and kidney function (urea, creatinine, uric acid, etc.) were recorded using an ERBA CHEM 7X instrument.

### 2.9 Histopathology

The organs were stored in 10% buffered formalin followed by dehydration using increasing alcohol concentrations (60%, 90%, and absolute) for 1 hour each. The tissue was embedded in paraffin wax after treatment with acetone and xylene. Thin sections measuring 4 microns in thickness were sliced using a microtome and subjected to hematoxylin and eosin staining. The sections were deparaffinized using xylene following hydration by treating with acetone and alcohol (5 min each). The sections were stained by dipping in hematoxylin solution followed by differentiating with acid alcohol. After treatment with eosin again, the sections were dipped in alcohol, acetone, and xylene before mounting with DPX. After drying, the slides were viewed under high-power light microscopy, and the observations were recorded by the expert pathologist. The photomicrographs of the tissue samples were taken at ×400 magnification.

### 2.10 Statistical analysis

The data were analyzed using GraphPad Prism 8 software and presented as means and standard deviation. Statistical tests including analysis of variance (ANOVA) were used to analyze differences among the groups, with statistical significance defined as *p* < 0.05.

## 3 Results

### 3.1 Characterization and measurement of nanoparticle sizes and size distributions

**FIGURE 1:**
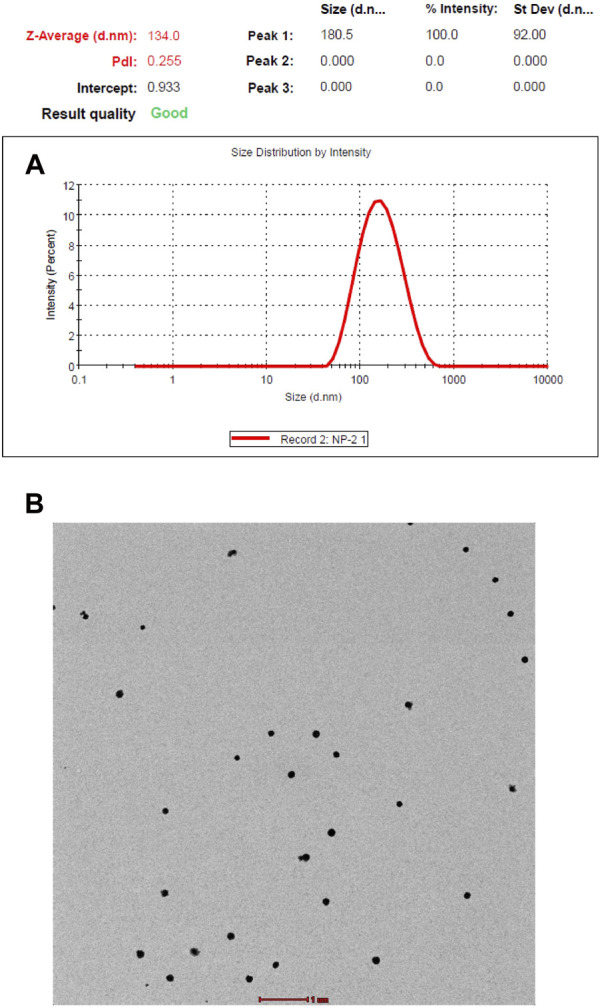
**(A)** Size distribution and polydisparity index of NIPAAM-VP-AA nanoparticles by dynamic light scattering (DLS). **(B)** Size and morphology of NIPAAM-VP-AA nanoparticles by transmission electron microscopy (TEM); scale bar = 1 µm.

The copolymerization of NIPAAM, VP, and AA resulted in the formation of amphiphilic polymers with hydrophobic cores and hydrophilic outer surfaces made of pyrrolidone, hydrated amides, and carboxylic groups. The hydrophobic core of the micelles made it suitable for accommodating any hydrophobic compound inside. The size and morphology were determined by DLS and TEM. DLS showed an average nanoparticle size of 134 nm with a polydispersity index of 0.27 at 25°C (Figure 1A). Transmission electron microscopy imaging showed that the synthesized polymers were spherical in shape, monodispersed, and 120 nm in size, comparable to the DLS measurements (Figure 1B). The drug (root extract) was loaded into the hydrophobic core of the micelles by simple vortexing and minor sonication.

### 3.2 FT-IR study

**FIGURE 2:**
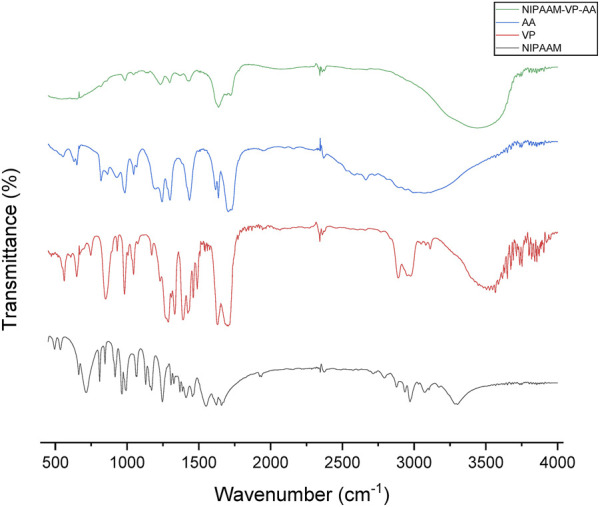
FT-IR spectra of N-isopropyl acrylamide (black), N-vinyl pyrrolidone (red), acrylic acid (blue), and NIPAAM-VP-AA polymeric (green) nanoparticles.

FT-IR spectra of the polymeric micelles and their monomers confirmed the polymerization in the reaction. The peaks of certain functional groups present in the spectra of the monomer units had disappeared or shifted in the polymeric nanoparticle spectra. For example, the spectrum of NIPAAM showed peaks between 2979.13 and 3272.50 cm^−1^ which confirmed the presence of aliphatic hydrocarbons. Peaks at 1659.19 cm^−1^ and 1720.67 cm^−1^ signified the presence of the carbonyl and C=C groups in the compound. These peaks were not particularly visible in the polymer spectra; the merged peaks of different functional groups were present in other monomers. The vinyl double bond peak at 800–1000 cm^−1^, amide peak at 1625.80–1681.82 cm^−1^, and amine peak at 3454.86 cm^−1^ in the vinyl pyrrolidone compound were also not reported in the spectrum of the polymer (Figure 2). Functional groups in the acrylic acid including the hydroxyl group, carbonyl, and the C=C group showed broad peaks at 3048.59–3430.88 cm^−1^, 1619.27 cm^−1^, and 1696.08 cm^−1^, respectively. The water of hydration attached to the polymer generated an intense peak at 3435 cm^−1^. Peaks at 2928 cm^−1^ and 1640–1720 cm^−1^ represented the stretching of the C-H and C=O bonds in the polymer, respectively. The manipulation in the peaks of different functional groups in the polymer clearly indicated that polymerization had been achieved.

### 3.3 GC-MS

**FIGURE 3:**
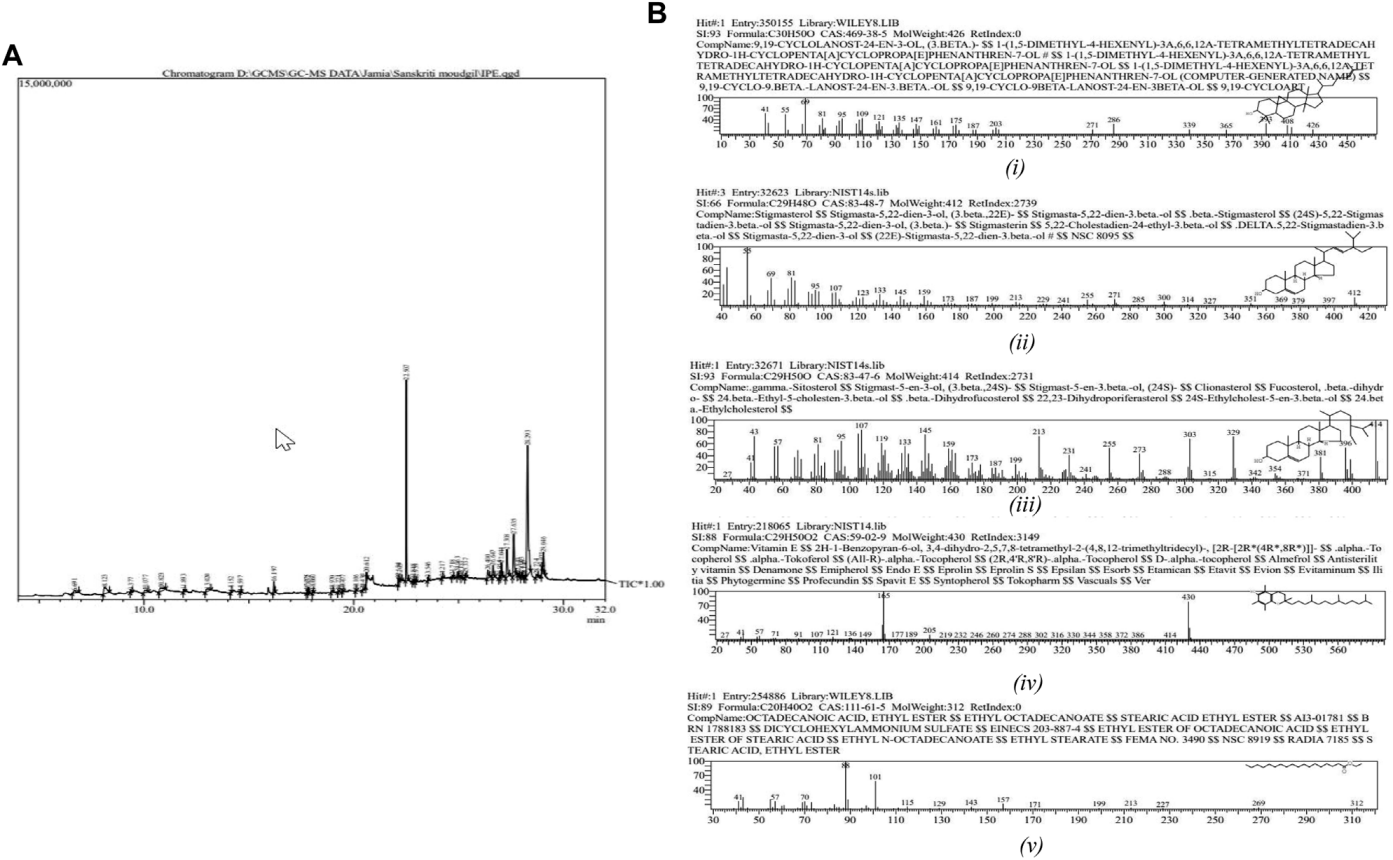
**(A)** Chromatogram of the GC-MS analysis of ethanolic root extract of *Ipomoea turpethum.* **(B)** Important compounds identified in the GC-MS analysis of the *Ipomoea turpethum* plant: i) 9,19-cyclolanost-24-en-3-ol, (3.beta.), ii) stigmasterol, iii) gamma-sitosterol, iv) vitamin E, and v) octadecanoic acid.

The GC-MS chromatogram of the ethanolic root extract of *I. turpethum* showed 42 volatile compounds present in the solution (Figure 3A; Table 1). The major constituents of the plant extract included sterols, organic acids, fatty acids, and tocopherols. 9,19-Cyclolanost-24-en-3-ol, (3.beta.) or cycloartenol (32.83%) was the most abundant compound, followed by terephthalic acid, bis(2-ethylhexyl) ester (19.95%), stigmasterol (8.91%), and sitosterol (6.16%). Other important compounds including vitamin E, octadecanoic acid, sitosterol, stigmasterol, campesterol, and palmitic acid were also identified. Some of these compounds were reported previously (Figure 3B) (Mughees and Wajid, 2020).

**TABLE 1:** Compounds identified by GC-MS analysis of an alcoholic root extract of *Ipomoea turpethum*.

Peak#	Retention time	Area	Area%	Name
1	6.691	477,697	0.93	12-Heptadecyn-1-ol
2	8.123	1,900,988	3.71	1H-Indene
3	9.377	201,145	0.39	N-Undecane
4	10.077	580,237	1.13	2-Butenedioic acid (E)-, bis(2-ethylhexyl) ester
5	10.823	1,292,256	2.52	Xanthosine
6	11.893	174,727	0.34	1-Pentadecene
7	13.020	677,790	1.32	1,2,3,5-Cyclohexanetetrol
8	14.152	84,684	0.17	1-Nonadecene
9	14.597	63,696	0.12	Neophytadiene
10	16.197	638,621	1.25	Palmitic acid and ethyl ester
11	17.775	157,563	0.31	Ethyl linoleate
12	17.832	81,458	0.16	9-Heptadecenoic acid, ethyl ester
13	18.060	144,941	0.28	Stearic acid, ethyl ester
14	18.970	161,417	0.31	Tritetracontane
15	19.271	105,583	0.21	1-Hexadecanal
16	19.477	107,151	0.21	4,8,12,16-Tetramethylheptadecan-4-olide (2)
17	20.106	78,349	0.15	Hexacosanal
18	20.430	120,566	0.24	Benzenepentanoic acid
19	22.203	179,956	0.35	Stearic acid, TMS derivative
20	22.507	10,224,372	19.95	Terephthalic acid, bis(2-ethylhexyl) ester
21	22.848	160,616	0.31	N-Tetracontane
22	22.959	164,895	0.32	Squalene
23	23.546	190,751	0.37	Tetratriacontane
24	24.217	129,514	0.25	Octadecanoic acid, ethyl ester
25	24.718	181,167	0.35	Gamma-Tocopherol
26	24.963	318,945	0.62	Stigmasterol acetate
27	25.133	57,268	0.11	Beta-sitosterol
28	25.337	278,635	0.54	Vitamin E
29	26.400	926,266	1.81	Campesterol
30	26.647	1,147,784	2.24	Stigmasta-5,22-dien-3-ol, (3.beta.,22e)-
31	26.954	109,350	0.21	Delta-9-tetrahydrocannabinol
32	27.044	1,431,901	2.79	4,14-Dimethylergosta-8,24(28)-dien-3-ol
33	27.308	3,159,069	6.16	.gamma.-Sitosterol
34	27.635	4,567,659	8.91	Stigmasterol
35	27.843	286,843	0.56	4-Campestene-3-one
36	28.003	497,399	0.97	Lupenone
37	28.147	140,432	0.27	Spinasterone
38	28.293	16,826,255	32.83	9,19-Cyclolanost-24-en-3-ol, (3.beta.)
39	28.724	447,769	0.87	3,11-Dihydroxy-12-ketocholanic acid
40	28.977	168,577	0.33	Stigmast-4-en-3-one
41	29.046	1,880,530	3.67	9,19-Cyclolanostan-3-ol, 24-methylene-, (3.beta.)-
42	42.746	728,538	1.42	Pentacosane

### 3.4 Body weight analysis

**FIGURE 4:**
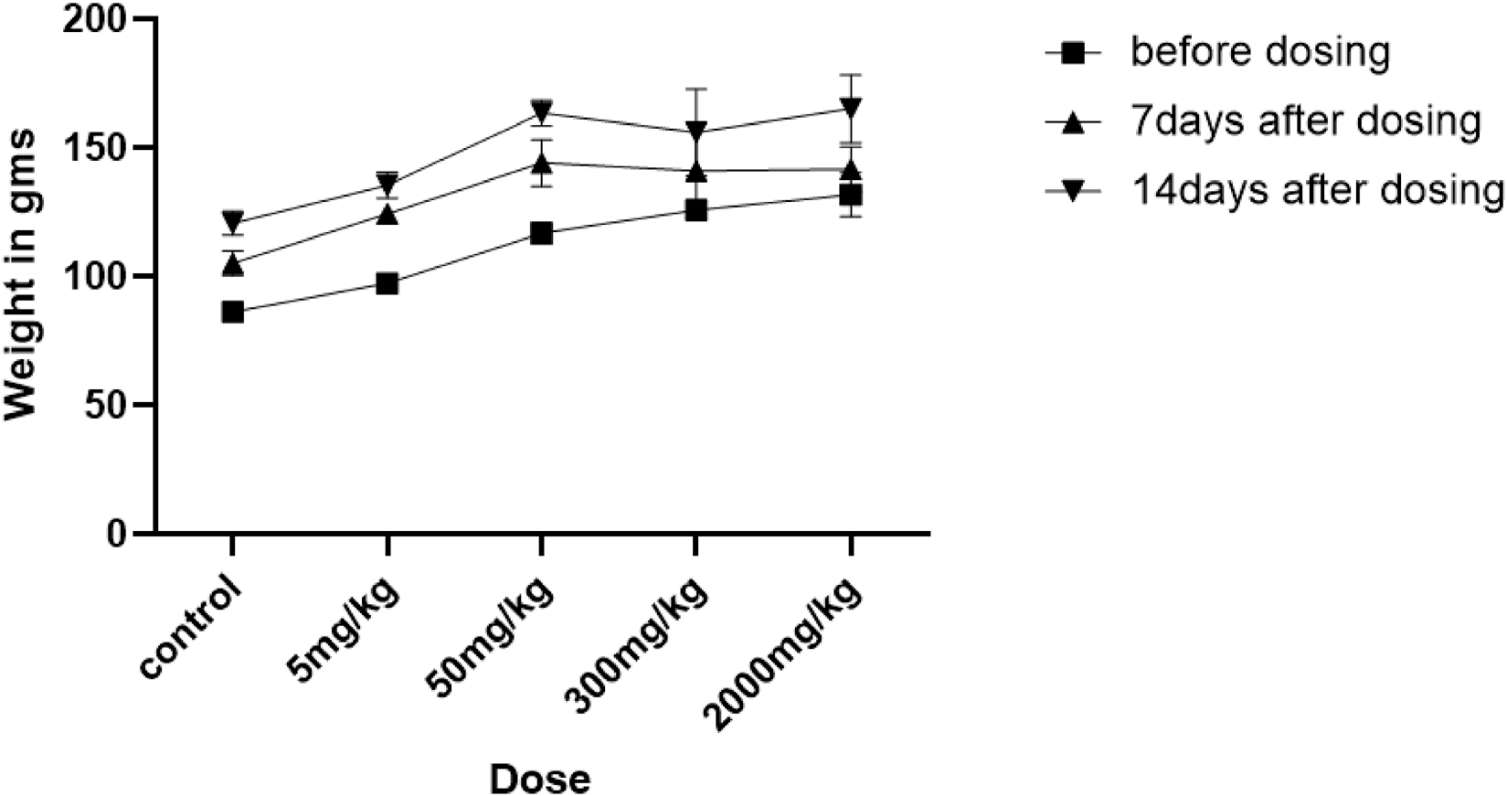
Changes in the weights of rats treated with different oral doses (5, 50, 300, and 2000 mg/kg and control) of *Ipomoea turpethum* extract loaded on polymeric nanoparticles at different time intervals (before dosing and 7 days after drug administration).

As reduced body growth can be a sign of toxicity, we measured the rats’ body weights before and after NVA-IT administration. Figure 4 shows the effect of the *I. turpethum* root extract-loaded nanoformulation on the mean body weights of the rats during the study. For the first 7 days, the two groups treated at the highest doses (300 mg/kg and 2000 mg/kg) did not show any significant weight gain compared to the other groups. The percentage weight gains of groups C and D were 12% and 7%, respectively, which were less than those of the other groups (21.86%, 27.79%, and 23.43% in control, group A, and group B, respectively). However, at the end of the second week, the animals showed more growth than the previous week. The other groups showed normal growth throughout the study (Supplementary Table S1) (**p < 0.05).

### 3.5 Behavioral study

After the dose was administered by oral gavage, the animals were kept under close observation for the first 30 min, and the changes in animal behavior were recorded. Later, the animals were observed at fixed time intervals, i.e., 1 h, 4 h, 8 h, 12 h, and then every 24 h for the next 13 days. The two highest-dosed animal groups showed some signs of toxicity including diarrhea, loss of appetite, and depression in the first 24 h of administration. Drowsiness was reported in the three treatment groups except for the lowest dose. No mortality was reported, suggesting that the drug was not lethal even at the highest dose (Supplementary Table S2).

### 3.6 Hematology and biochemical parameters

The hematology profile and biochemical parameters of the different groups were studied to detect drug toxicity in the blood, kidney, or liver. Hemoglobin, RBCs, and WBC count remained in the physiological ranges. Biochemical parameters including bilirubin, SGPT, SGOT, alkaline phosphatase, albumin, creatinine, urea, uric acid, and ion (Na^+^, Ca^2+^, and K^+^) levels in the treatment groups also did not differ significantly compared to those in the control group (Tables 2–4).

**TABLE 2:** Hematology profile of rats after 14 days of oral NVA-IT administration.

S. no	Parameter	Control	Group A	Group B	Group C	Group D
1	Hemoglobin (Hb) (gm/dL)	11.57 ± 1.4	11.87 ± 0.65	12.03 ± 1.24	13.2 ± 0.54	11.3 ± 1.26
2	TLC (total leukocyte count) (/cmm)	8,233.3 ± 3868	9,366.67 ± 3092	10,600 ± 1992	11,550 ± 3710.80	9,933.33 ± 3000
3	Differential leukocytic count (DLC)					
	Neutrophils (%)	7.63 ± 2.24	5.43 ± 4.1	10.23 ± 7.32	12.81 ± 7.28	7.74 ± 0.54
	Lymphocytes (%)	88.67 ± 2.11	91.6 ± 5.37	86 ± 9.06	83.25 ± 8.03	88.4 ± 1.25
	Eosinophils (%)	1.41 ± 0.21	1.05 ± 0.3	1.15 ± 0.50	1.46 ± 0.53	1.19 ± 0.49
	Monocytes (%)	1.80 ± 0.31	1.44 ± 0.47	2.22 ± 1.00	2.19 ± 0.69	1.86 ± 0.28
	Basophils (%)	0.48 ± 0.14	0.48 ± 0.3	0.41 ± 0.30	0.29 ± 0.04	0.72 ± 0.20
4	RBC (red blood cell count), (millions/cmm)	6.4 ± 0.54	6.01 ± 0.67	6.24 ± 0.64	6.74 ± 0.92	6.16 ± 0.86
5	PCV/HCT (hematocrit) (%)	38.43 ± 4.02	39.63 ± 2.15	40.73 ± 1.89	42.82 ± 1.44	38.5 ± 6.06
6	MCV (mean corpuscular volume) (fL)	60.03 ± 2.57	66.27 ± 5.26	65.57 ± 4.16	63.57 ± 7.23	62.4 ± 4.05
7	MCH (mean corpuscular Hb) (pg)	18.06 ± 0.72	19.87 ± 1.25	19.33 ± 1.44	19.6 ± 1.83	18.4 ± 0.80
8	MCH C (mean corpuscular Hb concentration) (gm/dl)	30.06 ± 0.87	29.93 ± 0.68	29.53 ± 2.81	30.85 ± 0.61	29.6 ± 1.53
9	Platelet count (lacs/cmm)	4.39 ± 2.55	5.26 ± 3.4	5.65 ± 1.24	8.61 ± 1.42	4.05 ± 3.77

**TABLE 3:** Liver function parameters in rats after 14 days of oral NVA-IT administration.

S. no.	Parameter	Control	Group A	Group B	Group C	Group D
1	Total bilirubin (mg/dl)	0.36 ± 0.07	0.62 ± 0.28	0.77 ± 0.33	0.56 ± 0.27	0.65 ± 0.53
2	Direct bilirubin (mg/dl)	0.12 ± 0.04	0.24 ± 0.13	0.45 ± 0.25	0.21 ± 0.11	0.25 ± 0.20
3	Indirect bilirubin (mg/dl)	0.24 ± 0.04	0.38 ± 0.16	0.62 ± 0.44	0.35 ± 0.18	0.40 ± 0.32
4	SGOT (U/L)	153.53 ± 43.45	231.2 ± 96.5	292.73 ± 152.94	151.20 ± 25.71	126.70 ± 27.70
5	SGPT (U/L)	47.63 ± 9.17	51.93 ± 5.52	61.93 ± 7.48	38.37 ± 3.00	52.43 ± 9.82
6	Alkaline phosphatase (U/L)	298.60 ± 80.03	344.28 ± 75.71	363.27 ± 199.19	247.40 ± 44.94	214.93 ± 163.88
7	Total protein (gm/dl)	6.36 ± 0.44	6.83 ± 0.86	7.89 ± 0.84	7.07 ± 0.74	6.45 ± 0.25
8	Albumin (gm/dl)	3.41 ± 0.46	3.72 ± 0.58	4.13 ± 1.04	3.71 ± 0.51	3.64 ± 0.32
9	Globulin (gm/dl)	2.95 ± 0.83	3.11 ± 0.84	3.76 ± 1.05	3.37 ± 0.31	2.81 ± 0.18

**TABLE 4:** Kidney function parameters in rats after 14 days of oral NVA-IT administration.

S. no.	Test	Control	Group A	Group B	Group C	Group D
1	Urea (mg/dl)	38.17 ± 7.10	30.37 ± 8.73	35.37 ± 6.89	40.53 ± 14.67	33.77 ± 1.53
2	Serum creatinine (mg/dl)	0.64 ± 0.08	0.66 ± 0.14	0.75 ± 0.04	0.71 ± 0.11	0.71 ± 0.21
3	Serum uric acid (mg/dl)	1.99 ± 0.69	2.54 ± 0.53	3.17 ± 1.61	2.45 ± 0.30	1.94 ± 0.29
4	Serum calcium (mg/dl)	9.19 ± 2.98	9.72 ± 2.76	10.22 ± 3.15	9.18 ± 0.66	9.98 ± 3.93
5	Sodium (mmol/L)	148.33 ± 2.08	136.5 ± 3.54	144.50 ± 0.71	157.33 ± 10.07	143.33 ± 3.51
6	Potassium (mmol/L)	5.14 ± 0.26	6.02 ± 0.17	8.15 ± 2.31	6.05 ± 1.09	5.38 ± 1.00
7	Chloride (mmol/L)	106.00 ± 3.46	103 ± 1.41	113.50 ± 3.54	113.67 ± 5.13	104.33 ± 5.69

### 3.7 Histopathological study

**FIGURE 5:**
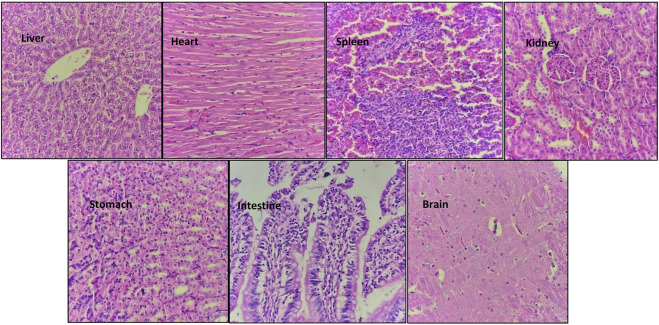
Hematoxylin and eosin-stained sections of vital organs harvested from Group D animals (2000 mg/kg) after 14 days of oral NVA-IT administration.

**FIGURE 6:**
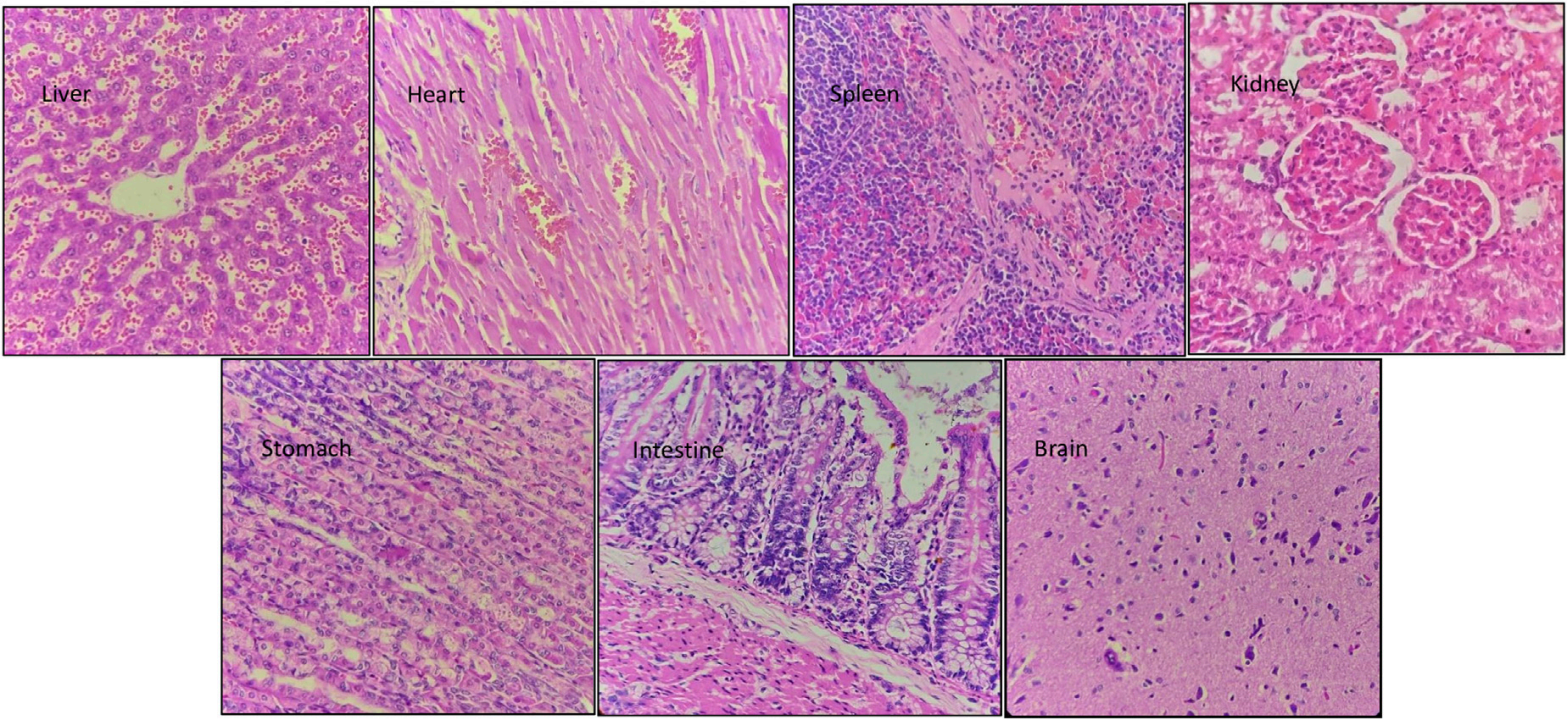
Hematoxylin and eosin-stained sections of vital organs harvested from Group C animals (300 mg/kg) after 14 days of oral NVA-IT administration.

Hematoxylin and eosin staining of the tissue sections was used to assess the cell morphology and arrangement in samples from the vital organs including the brain, heart, kidney, liver, spleen, stomach, and intestine. Groups C and D, the highest-dosed animals, did not show any anomalies related to the dose of NVA-IT. The sections of vital organs including the liver, heart, spleen, kidney, stomach, intestine, and brain showed no inflammation, morphological changes in the cells, or necrosis. The liver sections showed mild inflammatory infiltrates, and mild dilation of sinusoids along with RBC extravasation in a few areas, but the findings were not significant (Figures 5, 6). Lobular architecture, normal cell plate thickness, and polarity of the hepatic parenchyma were observed. The hepatocytes were polygonal in shape with well-formed nuclei and coarse chromatin. Overall, no inflammation, apoptosis, necrosis, biliary cell damage, inflammatory infiltrate, or fatty changes were observed in the liver sections of all groups (Supplementary Figures S1–S3). The heart sections showed normal myocyte arrangement, and the polarity was maintained in the cardiac muscle bundles in all groups. The intercalated disc was also conspicuous in all sections. The kidney sections showed cortex and medulla regions with the cortex demonstrating numerous glomeruli with normal capillary loops and mesangial matrix deposition. The proximal convoluted tubules, distal convoluted tubules, loop of Henle, and interstitium were normal. No areas of ischemia or necrosis were observed.

Examination of the spleen sections revealed white and red pulp showing primary lymphoid follicle formation and dilated sinusoids with the presence of RBCs, respectively. The brain sections also showed normal architecture of the glial components, i.e., astrocytes, oligodendrocytes, and axons accompanied by interspersed capillaries in a fibrillary background. The histological study of the stomach and intestine showed well-defined tissue structures with normal gastric mucosa comprising foveolar epithelial and glands with oxytocin, chief, and mucous cells in the stomach as well as normal intestinal mucosa, submucosa, and muscularis propria in the intestine. No inflammation or necrosis was observed in these organs.

## 4 Discussion

In this study, we successfully synthesized polymeric nanoparticles using NIPAAM, vinyl pyrrolidone, and acrylic acid as monomers, which provided temperature and pH sensitivity to the polymer. Fourier-transform infrared spectroscopy confirmed polymerization, with different peaks in the monomer samples compared to the polymerized sample. Some peaks disappeared and Some others represented the formation of new bonds. A nanoparticle diameter of nearly 100 nm was achieved during polymerization, with uniform morphology shown in DLS and TEM. Polymeric nanoparticles are best known for their property of retaining hydrophobic compounds in their core and releasing them in response to certain stimuli. NIPAAM exhibits a lower critical solution temperature (LCST) at nearly 31–33°C; beyond that range, the polymers get volume phase transition. This feature allows drug entrapment below LCST and release at physiological temperatures. By manipulating this nanoparticle property, we loaded the cores with the root extract of the *I. turpethum* plant. The combination of these three monomers provided a hydrophobic core made by PNIPAAM, pH sensitivity provided by acrylic acid, and temperature sensitivity imparted by vinyl pyrrolidone. These features make the nanoparticles double-triggered and allow them to reach active sites, such as cancer cells, intact, and collapsing occurs when there is a change in pH and temperature in the surrounding.

Our survey of the literature showed that *I. turpethum* has been used in both Unani and Ayurvedic systems of medicine since ancient times for the treatment of disorders including edema, anorexia, hepatosplenomegaly, hemorrhoids, fistula, worm infestation, anemia, abdominal tumors, and skin diseases (Semwal et al., 2019). Recent studies have reported its anti-inflammatory, anti-microbial, anti-ulcer, anti-diabetic, anti-diarrheal, anti-obesity, and anti-cancer activity. *I. turpethum* also works as an analgesic and CNS depressant and shows laxative and hepatoprotective effects (Sudan et al., 2016). The GC-MS data of ethanolic extract of *I. turpethum* roots revealed 42 compounds, including terephthalic acid, xanthosine, palmitic acid, beta-sitosterol, stigmasterol, and vitamin E. Some of the compounds have also been reported in a previous GC-MS analyses of the *I. turpethum* plant, which authenticates our plant specimen. For instance, Mughees and Wajid (2020) identified 71 compounds in the plant, some of which were also identified in our GC-MS, including 1H-indene, ethyl linoleate, octadecanoic acid, stigmasterol, β-sitosterol, campesterol, and palmitic acid. Two compounds, palmitic acid and n-undecane, were identified by Hamedi et al. (2014). Compounds like β-sitosterol and stigmasterol can induce apoptosis and immune modulation and have anthelminthic and anti-diabetic effects, which clearly demonstrate the ancient use of the plant in different diseases (Saeidnia et al., 2014). Palmitic acid shows anti-inflammatory and anti-tumor activity by suppressing the PI3/AKT pathway (Zhu et al., 2021). Vitamin E is well known for its antioxidant, neuroprotective, immune-boost, and anemia-curing effects (Jilani and Iqbal, 2018; Meydani et al., 2018; Lee and Ulatowski, 2019). Cycloarterol or 9,19-cyclolanost-24-en-3-ol, (3.beta.) shows antimicrobial and anti-cancer activities. This compound decreases the proliferative activity in cancer cells by cell cycle arrest and triggering cell apoptosis (Niu et al., 2018). The GC-MS analysis of the ethanolic root extract of *I. turpethum* assisted in the identification of the medicinal potential of the plant. Furthermore, new compounds were identified, including delta-9-tetrahydrocannabinol, which was not reported previously.

The results of the acute oral toxicity study suggested that the polymeric nanoparticles loaded with *I. turpethum* extract were non-toxic even at the highest dose (2000 mg/kg); thus, they can be allocated to category 5 under the Globally Harmonized System of Classification and Labeling of Chemical (GHS). As female Wistar rats are more sensitive than male Wistar rats, they were chosen for this study. The rats were divided into groups and administered different doses in a stepwise manner, i.e., vehicle, 5 mg/kg, 50 mg/kg, 300 mg/kg, and 2000 mg/kg by oral gavage. No toxicity symptoms were observed for the lowest dose whereas the rest showed some drowsiness immediately after dosing. No motor activity effects or toxicity symptoms such as a change in skin color, piloerection, and anxiety were observed in any group. However, the two highest doses showed a loss of appetite, diarrhea, and depression within 24 h, but these symptoms lasted only for 48 h. Weight gain was less in the groups administered 300 mg/kg and 2000 mg/kg doses compared to the groups administered 5 mg/kg, 50 mg/kg, and control after 1 week of NVA-IT treatment. However, the weight gain was normal in all the groups at end of 2 weeks. No mortality was reported in any group. Thus, the LD_50_ value of NVA-IT was determined to be >2000 mg/kg. The literature has reported similar results regarding the toxicity status of *I. turpethum* plant extract. In their acute oral toxicity study in mice, Khan et al.classified *I. turpethum* as slightly toxic, with an LD_50_ value of 0.5–5.0 gm/kg, whereas Sharma et al. reported no lethality up to a dose of 2000 mg/kg in Wistar rats. Another study reported an LD_50_ of *I. turpethum* methanolic plant extract of 1917.66 mg/kg in mice (Sharma and Singh, 2012; Nayeem et al., 2016; Gupta and Ved, 2017).

Blood parameters are indicators of physiological and pathological changes in the body of an organism due to the sensitivity of hematopoietic cells to xenobiotics (organic/synthetic/radiation). These can indicate not only the detrimental effect of the substance on the blood but also the blood-related function of the drug. The NVA-IT nanoparticles did not alter blood parameters including RBCs, Hb, and PCV; thus, the metabolites did not promote anemia, hemorrhage, or hemolysis in the rats and the balance between the rate of formation and death of blood cells was unaffected. The lack of a significant difference in MCV, MCH, and MCVC levels between the treated and control groups showed that the incorporation of hemoglobin in the RBC was normal. The CBC results indicated that NVA-IT did not affect the morphology, osmotic fragility, or the Hb-incorporating ability of RBCs in the blood (Adebayo et al., 2010; Owolarafe et al., 2020). White blood cells represent the immune system of the body; an increase in leukocyte count is triggered by the invasion of a toxic substance or pathogen. In our study, the total leukocyte count was slightly but not significantly enhanced in animals treated with different doses of NVA-IT compared to the control group. The increase in TLC is the normal defensive response of the immune system toward foreign substances. However, the differential count did not differ significantly, suggesting the non-allergic and harmless nature of the administered substance to the immune system.

Clearing the toxic substances from the blood is one of the most important roles of the liver; thus, any toxic effect of a drug is reflected by liver function. SGOT/AST and SGPT/ALT are the indicators of liver injury that cause the release of these enzymes in the blood. Albumin and globulin protein in the serum reflect the synthetic function of the liver, whereas bilirubin indicates the excretory function. Hepatocellular injury and toxicity are indicated by changes in protein and bilirubin levels. In our study, we observed no significant difference in the concentrations of liver enzymes, total protein, albumin, and bilirubin in all the groups, which indicated that NVA-IT did not affect the normal functioning of the liver (Yakubu et al., 2012). The kidney maintains the body’s homeostasis by controlling the electrolyte and fluid levels and removing toxins and waste from the body. The serum urea and creatinine are indicators of glomerular function, while the serum electrolyte level represents renal tubule function. The normal levels of urea, creatinine, and electrolytes in all the groups in this study suggested that the nanoparticles loaded with *I. turpethum* ethanolic root extract did not hamper the normal body homeostasis and did not lead to kidney damage or renal tubular stress in the rats. The vital organs, such as the brain, heart, liver, spleen, kidney, stomach, and intestine, subjected to histopathological examination showed normal cell architecture in both the control and treatment groups. The histopathology findings corresponded to those of the biochemical study of the blood, which confirmed our inferences (Owolarafe et al., 2017). The stomach and intestine sections showed normal histology, indicating that the formulation did not corrode the gastric or intestinal epithelia.

Many studies have reported the various curative uses of *I. turpethum* extract. Pulipaka et al. demonstrated the anti-diabetic effect of a methanolic root and stem extract in streptozotocin-induced diabetic rats. The anti-inflammatory effect in formalin-induced rat paw edema and GIT-related disorder by *I. turpethum* extract or formulation has also been reported (Bhande et al., 2006; Kumar et al., 2006; Pulipaka et al., 2012; Tveden-Nyborg et al., 2012)*.* The anti-cancer activity of *I. turpethum* extract was documented in oral squamous cell carcinoma by Arora et al. and in breast cancer cell lines by Mughees (Arora et al., 2017; Mughees et al., 2019). Loading the extract into polymeric nanoparticle core will enhance the extract potency and stability and specifically target cancer cells due to the double-triggered (pH and temperature) property of the nanoparticles. This formulation can be used for the treatment of the previously mentioned conditions after thorough study in disease animal models. Among future prospects, this formulation can be tested in different animal cancer models to assess the cumulative effects of *I. turpethum* extract delivered *via* polymeric nanoparticles.

## 5 Conclusion

The acute oral toxicity study has inferred that the polymeric nanoparticles loaded with *Ipomoea turpethum* extract are non-toxic even at the highest dose i.e., 2000 mg/kg, and can be allocated to category 5 under the Globally Harmonized System (GHS) of Classification and Labeling of Chemical. The biochemical parameters and the histoarchitecture of the vital organs did not show any sign of toxicity. Thus, we conclude that the formulation can be used as a therapeutic agent for deadly diseases such as cancer without any harmful effect on the other normal cells of the body.

## Data Availability

The original contributions presented in the study are included in the article/Supplementary Material. Further inquiries can be directed to the corresponding author.

## Abbreviations

NIPAAM, N-isopropyl acrylamide; VP, N-vinyl pyrrolidone; AA, acrylic acid; NVA-IT, *Ipomoea turpethum* extract-loaded (NIPAAM- N-vinyl pyrrolidone-acrylic acid) polymeric nanoparticle; GC-MS, gas chromatography-mass spectrometry; TEM, transmission electron microscopy; DLS, dynamic light scattering; FT-IR, Fourier-transform infrared spectroscopy.
