# Supplementary material for: Evaluation of acute oral toxicity of Ipomoea turpethum extract loaded polymeric nanoparticles in Wistar rats
Source: Front Pharmacol. 2023 Mar 15;14:1086581. doi: 10.3389/fphar.2023.1086581 (PMC10050689; doi:10.3389/fphar.2023.1086581)
Supplement: Supplementary file 1 [file DataSheet1.PDF]

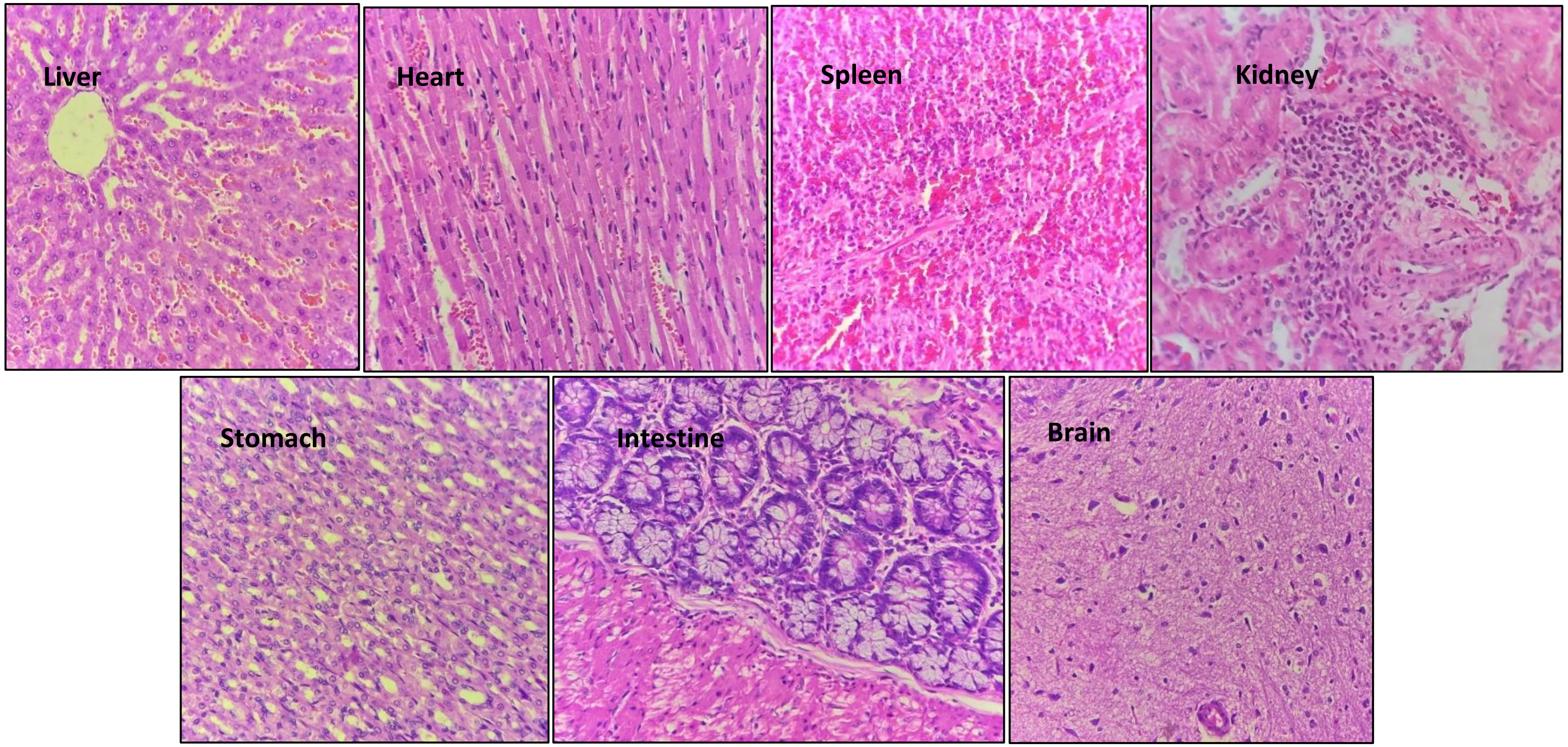

Figure S1 Haematoxylin and Eosin stained sections of vital organs harvested from Group B animals (50mg/kg) after 14 days of oral NVA-IT administration.

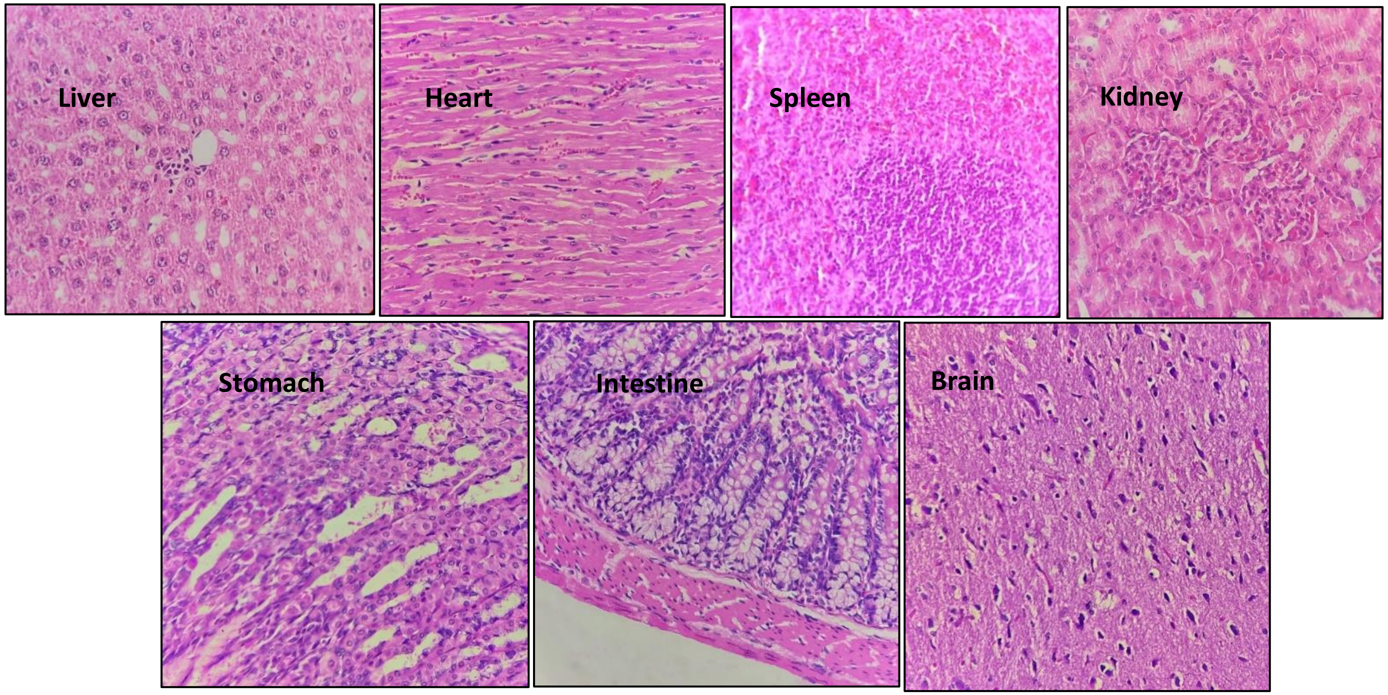

Figure S2 Haematoxylin and Eosin stained sections of vital organs harvested from Group B animals (5mg/kg) after 14 days of oral NVA-IT administration.

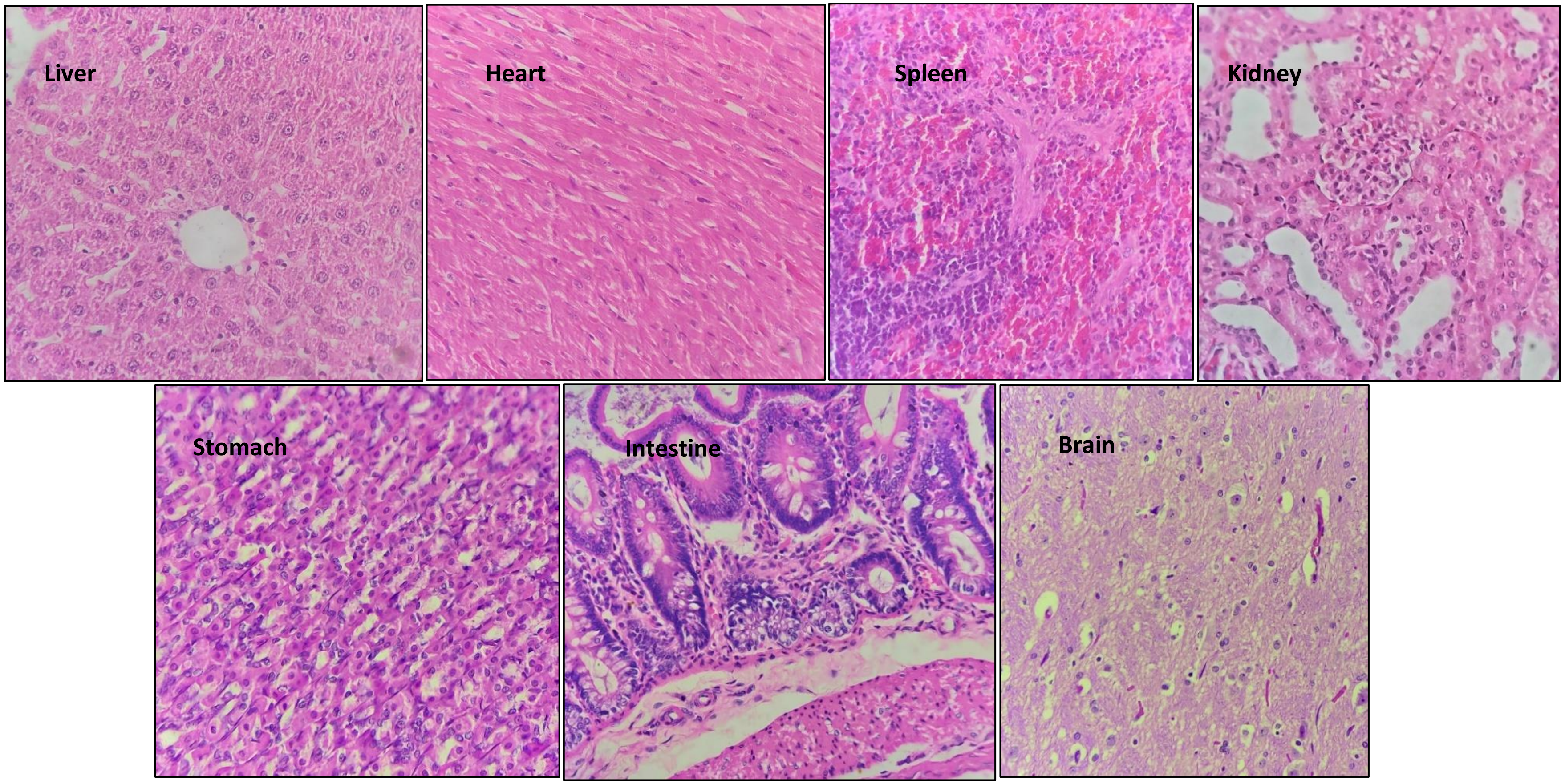

Figure S3 Haematoxylin and Eosin stained sections of vital organs harvested from Group A animals (vehicle) after 14 days of oral vehicle administration.

Table S1 Percentage weight gain of animals after oral administration of NVA-IT nanoparticles at the period of 7 days and 14 days.

Time Duration	Control	Group A	Group B	Group C	Group D
7 days	21.86%	27.79%	23.43%	12.07%	7.47%
14 days	39.84%	39.28%	40%	24.01%	25.32%

Table S2 Behavioural study of Animals in the first 24 hrs after the oral NVA-IT administration.

Behavioural Changes (after dosing)	Control	Group A (5mg/kg)	Group B (50mg/kg)	Group C (300mg/kg)	Group D (2000mg/kg)
Decrease of motor activity	N	N	N	N	N
Increase of motor activity	N	N	N	N	N
Loss of reflections or straightening	N	N	N	N	N
Change in the skin	N	N	N	N	N
Erection of the tail	N	N	N	N	N
Piloerection	N	N	N	N	N
Drowsiness	N	N	P	P	P
Diarrhoea	N	N	N	P	P
Aggressive	N	N	N	N	N
Afraid	N	N	N	N	N
Death	N	N	N	N	N
Weight variation	N	N	N	P	P
Red eye	N	N	N	N	P
Depressed	N	N	N	P	P
Anxiety	N	N	N	N	N
Loss of appetite	N	N	N	P	P
